# Supplementary material for: Divergent disruption of brain networks following total and chronic sleep loss: a longitudinal fMRI study
Source: Sleep. 2026 Feb 3;49(5):zsag030. doi: 10.1093/sleep/zsag030 (PMC13163182; doi:10.1093/sleep/zsag030)
Supplement: Supplementary_materials_clean_zsag030 [file supplementary_materials_clean_zsag030.docx]

# **Supplementary materials**

**Divergent disruption of brain networks following total and chronic sleep loss:
a longitudinal fMRI study**

**Authors:**

Patrycja Scislewska^1^, Arturo Cabrera Vazquez^2,3,4^, Iwona Szatkowska^5^,

Halszka Kontrymowicz-Ogińska^6^, Sophie Achard^2 #^*, Aleksandra Domagalik^7 #^*

* These authors contributed to this work equally.

# Corresponding authors:
sophie.achard@univ-grenoble-alpes.fr
aleksandra.domagalik@uj.edu.pl

**Affiliations:**

^1^ University of Warsaw, Faculty of Biology, Institute of Experimental Zoology, Warsaw, Poland

^2^ Univ. Grenoble Alpes, CNRS, Inria, Grenoble INP, LJK, 38000 Grenoble, France

^3^ Univ. Grenoble Alpes, Inserm, U1216, Grenoble Institut Neurosciences, 38000 Grenoble, France**.**

^4^ INSERM U1214, Toulouse Neuroimaging Center, CHU Purpan, 31059 Toulouse, France

^5^ Laboratory of Emotions Neurobiology, Nencki Institute of Experimental Biology, Polish Academy of Sciences, 02-093 Warsaw, Poland

^6^ Department of Cognitive Neuroscience and Neuroergonomics, Institute of Applied Psychology, Jagiellonian University, 30-348 Kraków, Poland

^7^ Centre for Brain Research, Jagiellonian University, 31-501 Kraków, Poland

#

S1. Modified version of AAL atlas description. Text reproduced from [[39]](https://www.zotero.org/google-docs/?TBS8tz), Supplementary Materials:

Modified version of classical AAL parcellation scheme The classical AAL parcellation scheme is composed by 116 regions including the cerebellum. We have merged some of the regions, reducing the parcellation to 89 regions. Merged regions are: frontal medial orbital and rectus (one region for left and one for right hemisphere); occipital superior, middle and inferior (one region for left and one for right hemisphere); temporal pole superior and medial (one region for left and one for right hemisphere); the cerebral crus (one region for left and one for right hemisphere); areas III, IV, V and VI of cerebellum (one region for left and one for right hemisphere); areas VII, VIII, IX, X of cerebellum (one region for left and one for right hemisphere) and finally, the vermis (one single region for both hemispheres).

S2. Quality check of the fMRI data after motion regression (following the approach of Power and colleagues [[41]](https://www.zotero.org/google-docs/?mMTL83)). A) For each frame of data in one subject, the framewise displacement (FD) of a frame of data is plotted against the absolute values of the differentials of RS fMRI timecourses of 89 ROIs. A locally weighted regression (LOESS) curve (black line) was fitted to the relationship between FD and BOLD signal change using a span parameter of 0.02, enabling the visualization of fluctuations and potential motion-related artifacts. B, C, D) Identically produced LOESS curves from all 28 subjects in all three scanning sessions are plotted against FD. These results confirm that there is no significant relationship between motion and the BOLD signal in the described dataset.


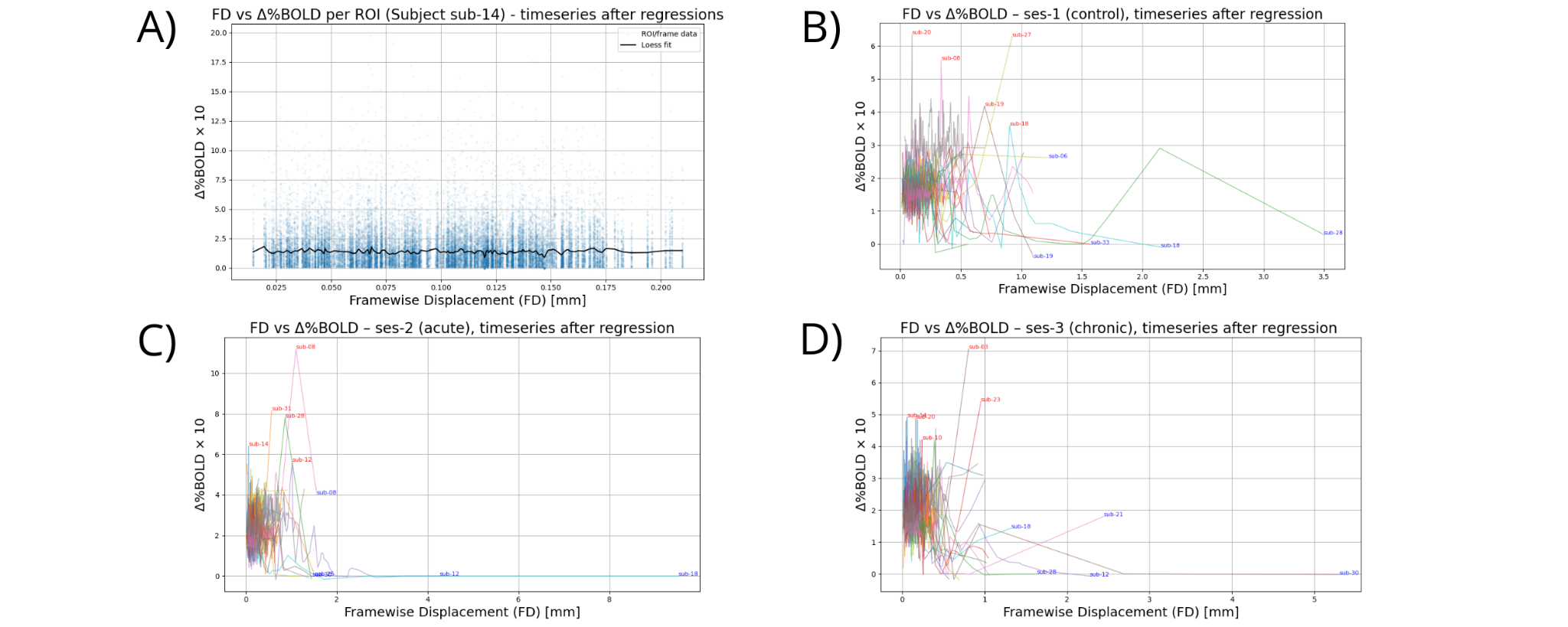


S3. Boxplots with I) numbers of significant edges per participant per scanning session for both atlases (modified AAL and AICHA). Dots represent participants. Each plot is annotated by the subject’s ID with the smallest number of significant edges, II) global mean correlation values from wavelet scale 3 per participant per scanning session for both atlases (modified AAL and AICHA).


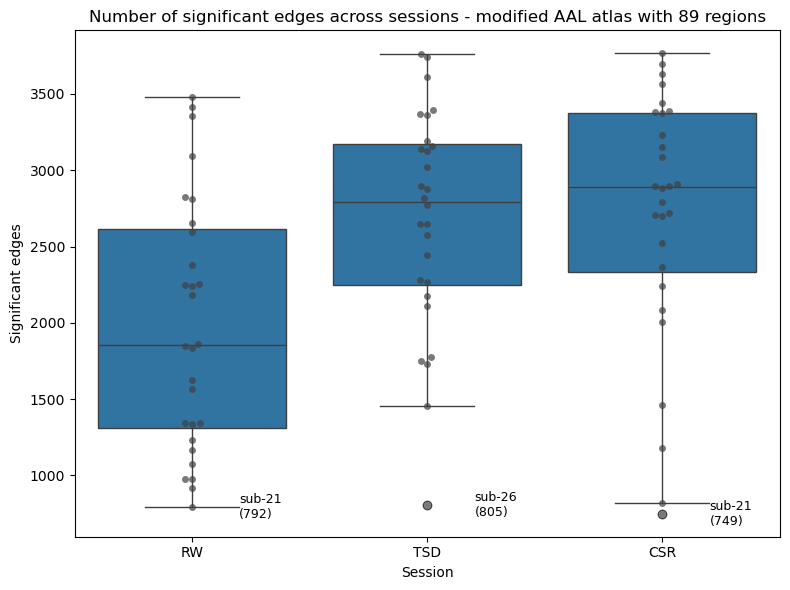


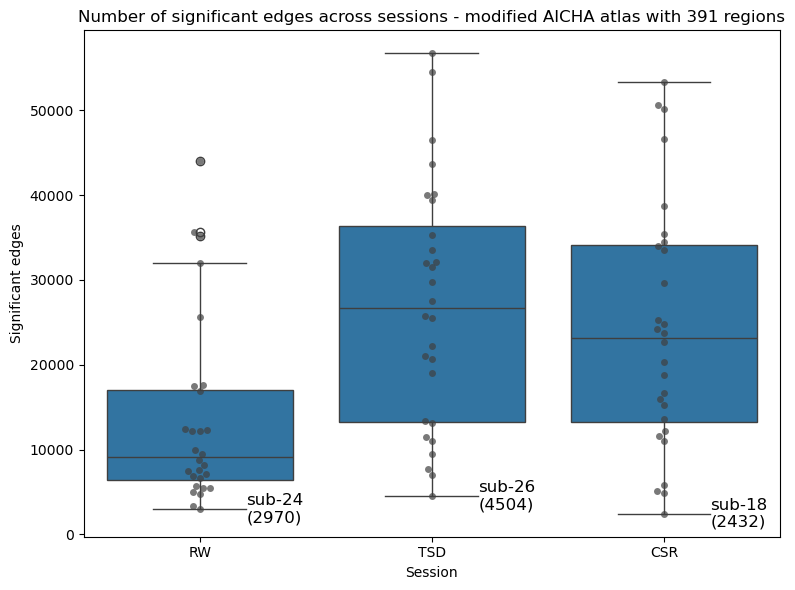


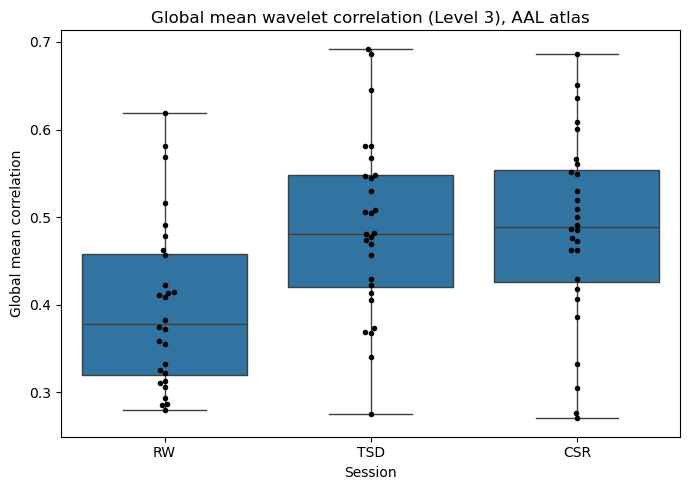


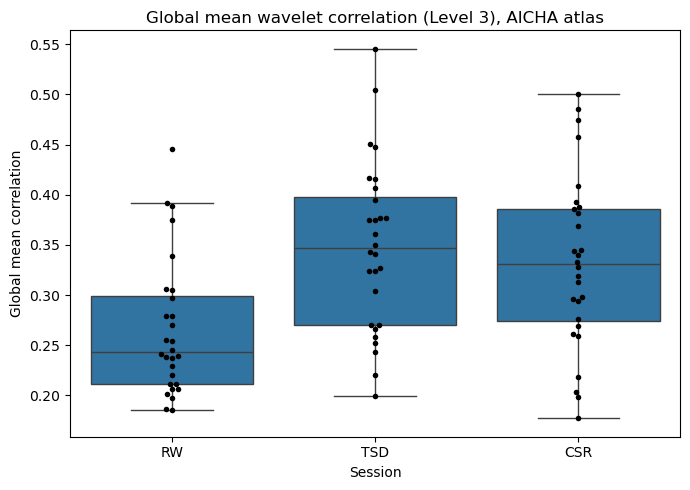


S4. Equations for nodal metrics:

The simplest graph metric is the degree of a node *i*, D*_i_*. It corresponds to the number of edges connected to a node *i*

$$D_{i}= \sum_{j} a_{ij}$$

where $a_{ij}$​ is the entry in the adjacency matrix *A*, with $a_{ij}=1,$ if nodes *i* and *j* are connected, and 0 otherwise. Degree centrality is normalized by dividing degree value by the highest node degree in the network.

We also tested the clustering coefficient Cc_i_, which quantifies how nodes in a graph tend to cluster together and form local groups [[52]](https://www.zotero.org/google-docs/?MzbFKY). The clustering coefficient is defined as the ratio between twice the number of connections among neighbors of node *i* and the total number of possible connections between those neighbors.

$$Cc_{i}= \frac{2t_{i}}{D_{i}(D_{i}-1)}$$

where *t_i_* is the number of connections among neighbors of node *i.*

The third considered metric is closeness centrality, which reflects how quickly the information can be spread through the network and is calculated as the inverse of the average shortest distance from the node to all other nodes in the graph. [[51]](https://www.zotero.org/google-docs/?wYBkzT).

$$C_{i}= \frac{1}{\sum_{i\neq j} d_{ij}}$$

where d_ij_ is the shortest path distance between nodes *i* and *j.*

S5. Schematic representation of permutation tests performed to evaluate the within-subject HDI method. A) Original data: participants scanned under different conditions (here: reference condition (marked in orange) and condition *t* (marked in blue)) with correct within-subject pairing and condition labels. Dots represents the nodes, color code (light red, vivid red, dark red) represents example degree values. B) Node permutations: within-subject pairing and condition labels are preserved, but degree of nodes (here: colors of nodes) is randomly shuffled in the condition *t* scans. C) Unpaired permutations: correct condition labels, but participants' order in the condition *t* scans is randomly mixed across all subjects, breaking within-subject pairing. D) Paired permutations: condition labels are randomly assigned, preserving the pairing but not the original condition assignments.


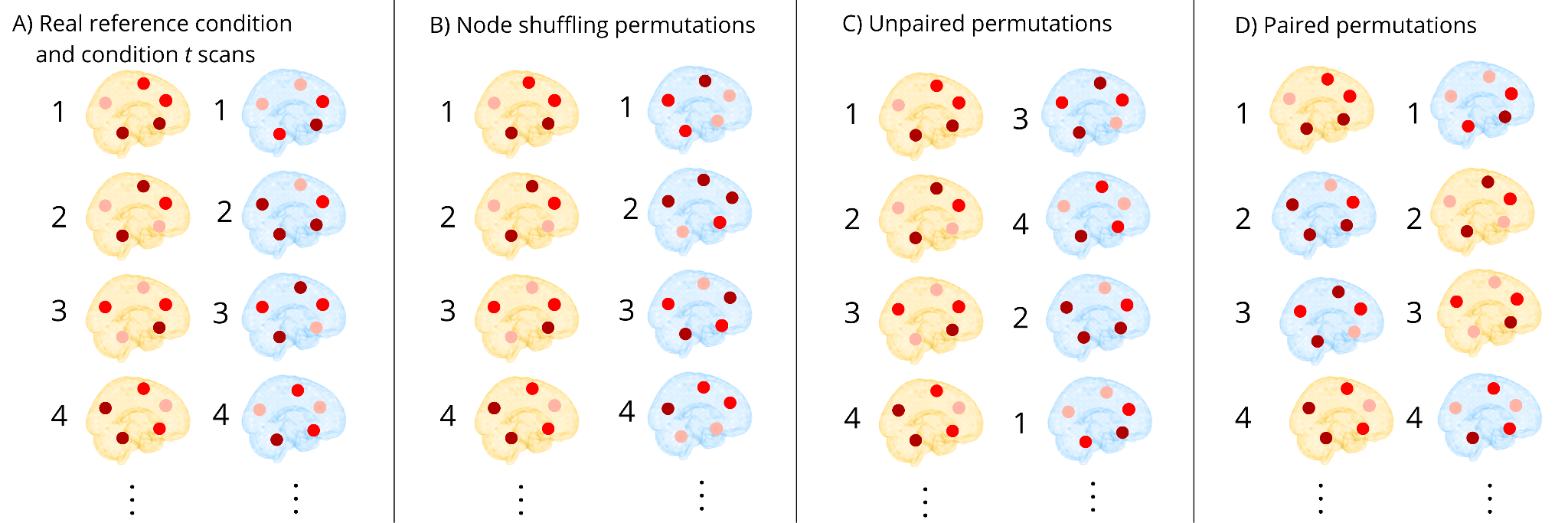


S6. Results of the Node shuffling permutations test and Unpaired permutations test.


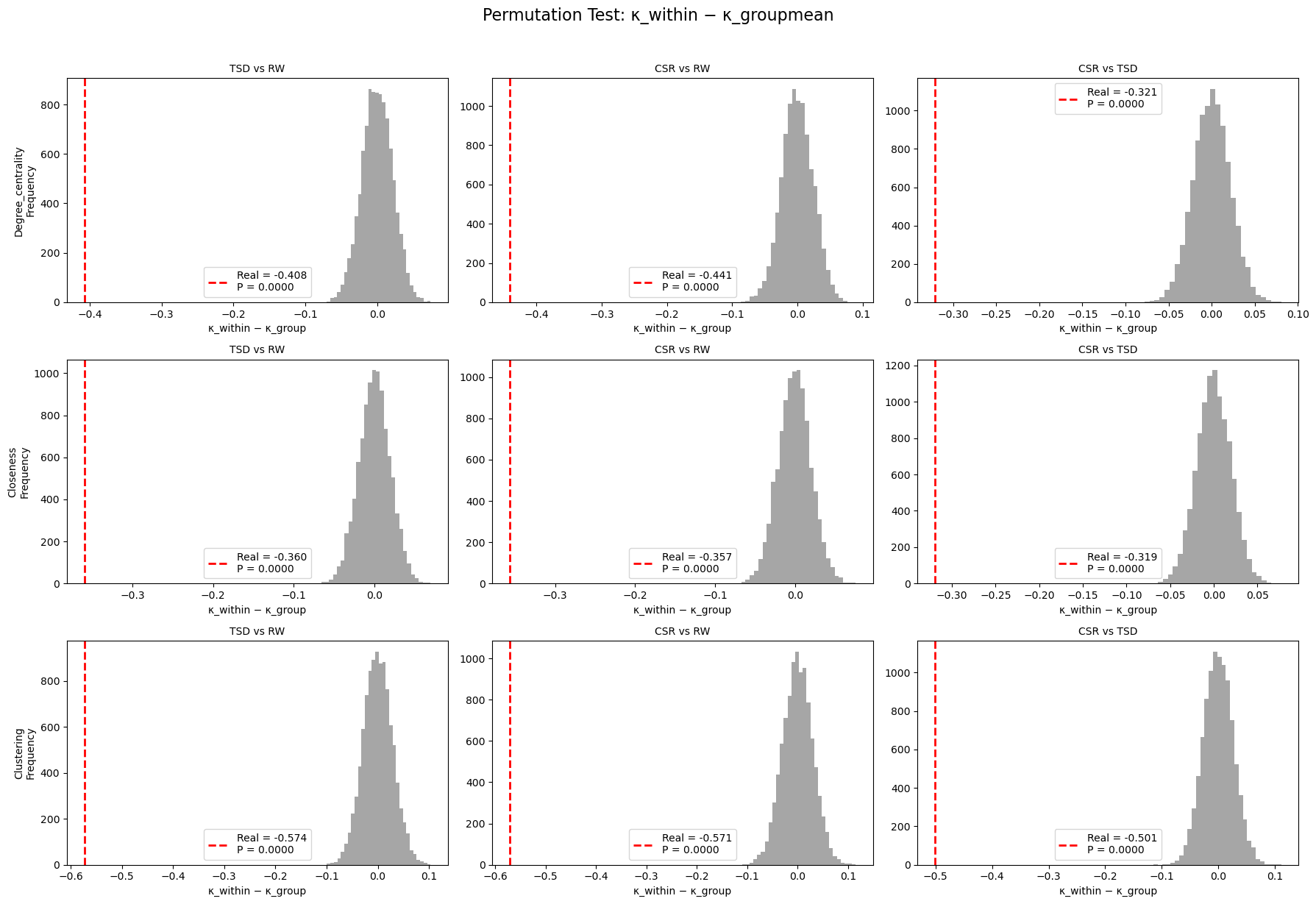


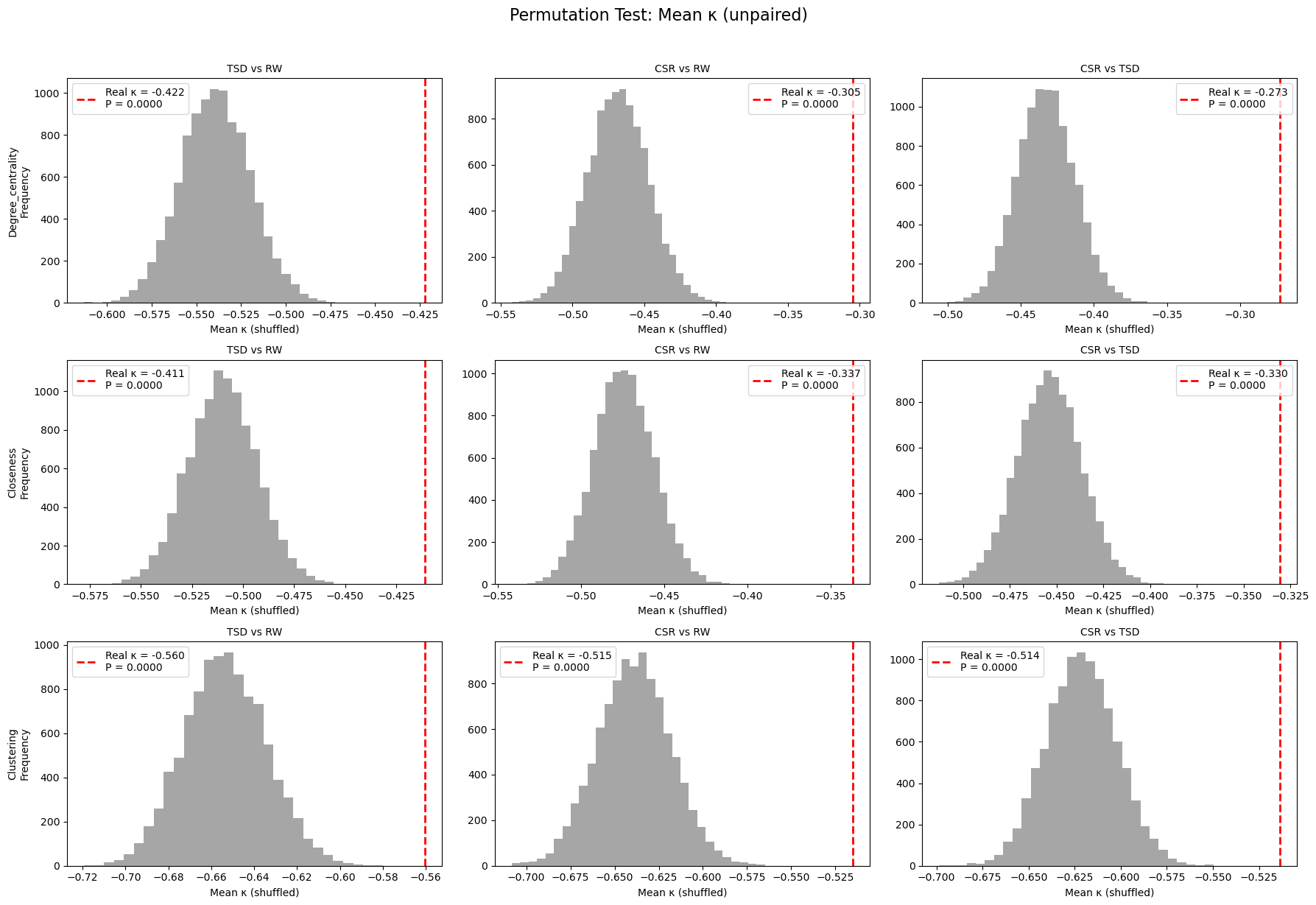


Results of Paired permutations test are provided in the main text, Figure 5.

S7. Table with details from linear mixed-effects model for global metrics. Bolded and underlined text indicates significant results (p < 0.05), bolded text indicates trend-level associations.

| **Metric** | **Comparison** | **Estimate** | **CI Lower Bound** | **CI Upper Bound** | ***p*-value** | ***p*-value (FDR)** |
| --- | --- | --- | --- | --- | --- | --- |
| **Global efficiency** | TSD vs RW | -0,0048 | -0,0124 | 0,0028 | 0,2186 | 0,2643 |
|  | CSR vs RW | 0,0003 | -0,0073 | 0,0079 | 0,9364 | 0,9364 |
|  | CSR vs TSD | 0,0051 | -0,0025 | 0,0127 | 0,1901 | 0,1901 |
| **Average clustering** | TSD vs RW | 0,0085 | -0,0061 | 0,0230 | 0,2528 | 0,2643 |
|  | CSR vs RW | -0,0074 | -0,0220 | 0,0071 | 0,3156 | 0,7890 |
|  | **CSR vs TSD** | **-0,0159** | **-0,0305** | **-0,0014** | **0,0318** | **0,0530** |
| **Average path length** | TSD vs RW | 0,0805 | -0,0382 | 0,1991 | 0,1838 | 0,2643 |
|  | CSR vs RW | -0,0056 | -0,1243 | 0,1130 | 0,9259 | 0,9364 |
|  | CSR vs TSD | -0,0861 | -0,2047 | 0,0326 | 0,1550 | 0,1901 |
| **Modularity** | TSD vs RW | 0,0151 | -0,0114 | 0,0416 | 0,2643 | 0,2643 |
|  | CSR vs RW | -0,0224 | -0,0489 | 0,0041 | 0,0971 | 0,4856 |
|  | **CSR vs TSD** | **-0,0375** | **-0,0640** | **-0,0110** | **0,0055** | **0,0276** |
| **Average graph distance** | TSD vs RW | 0,2336 | -0,0054 | 0,4727 | 0,0554 | 0,2643 |
|  | CSR vs RW | -0,0364 | -0,2755 | 0,2027 | 0,7653 | 0,9364 |
|  | **CSR vs TSD** | **-0,2701** | **-0,5091** | **-0,0310** | **0,0268** | **0,0530** |

S8. Permutation tests to check the robustness of results of the linear mixed-effects model analysis for global metrics.
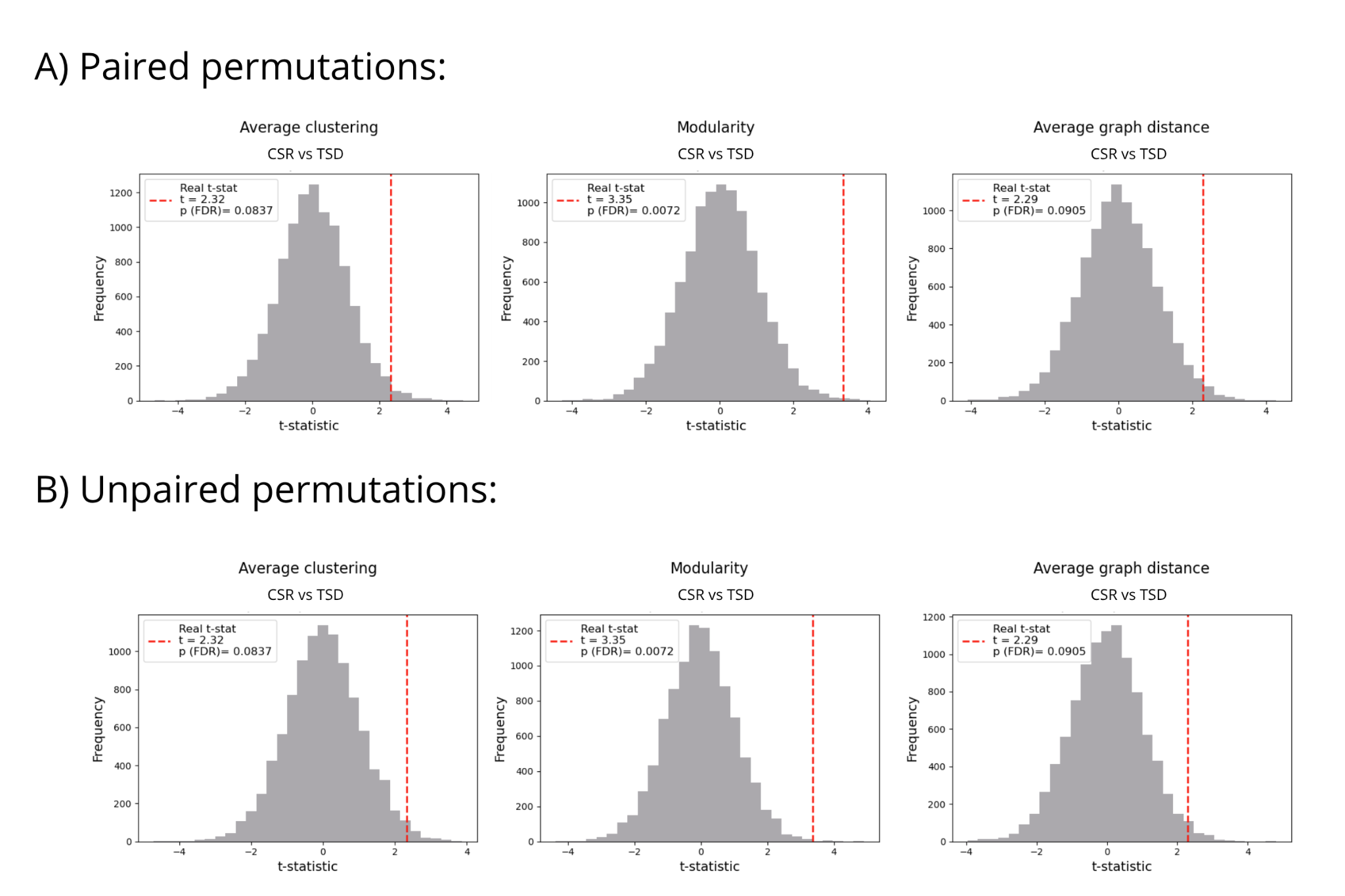


S9. Connectograms of the 28 participants in our cohort.

RW TSD CSR RW TSD CSR RW TSD CSR


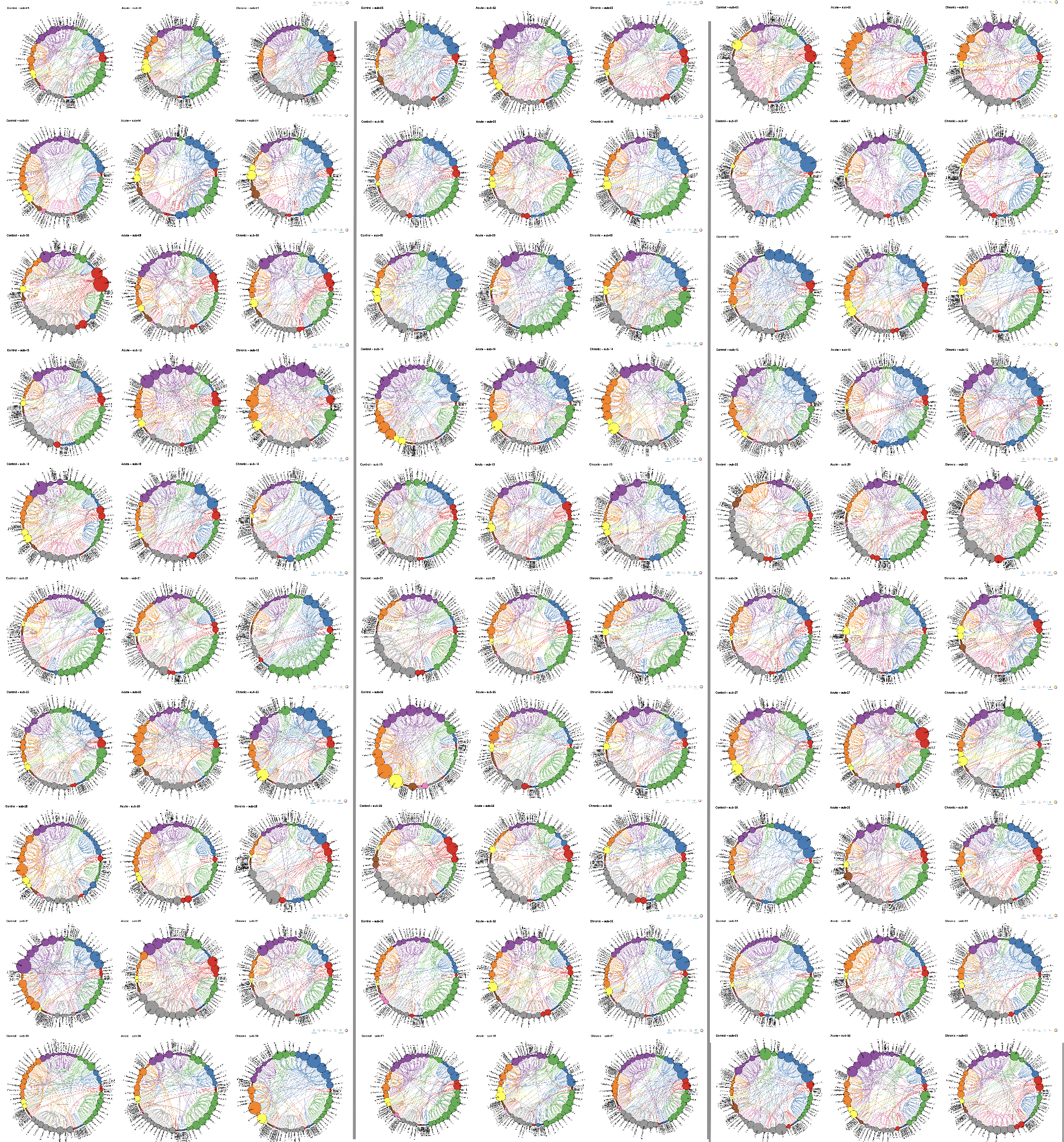


S10. Detailed description of CCML optimization strategy and results of the CCML analysis based on closeness centrality.

The reduced point *x* ̃_i_ is defined by *x* ̃_i_ = [*αc*_i_; *x*_i_]*^T^*, where *c*_i_ is the chosen covariate (in this study: κ value) and *x*_i_ represents the other coordinates in the embedding space. A scaling factor *α* is introduced to balance the covariate axis with the rest of the dimensions. Factor *α* is estimated by optimization. We initialized the *α* parameter based on the relative spread of κ values and the initial ISOMAP embedding. To improve embedding stability, we selected the first two subjects as the most distant pair based on ISOMAP geodesic distances and then iteratively added the subject that was farthest from those already embedded. CCML then sequentially optimized each subject’s position in embedding by minimizing the discrepancy between geodesic distance and the distance in combined network-covariate space, followed by global refinement of both subject coordinates and the *α* scaling parameter.


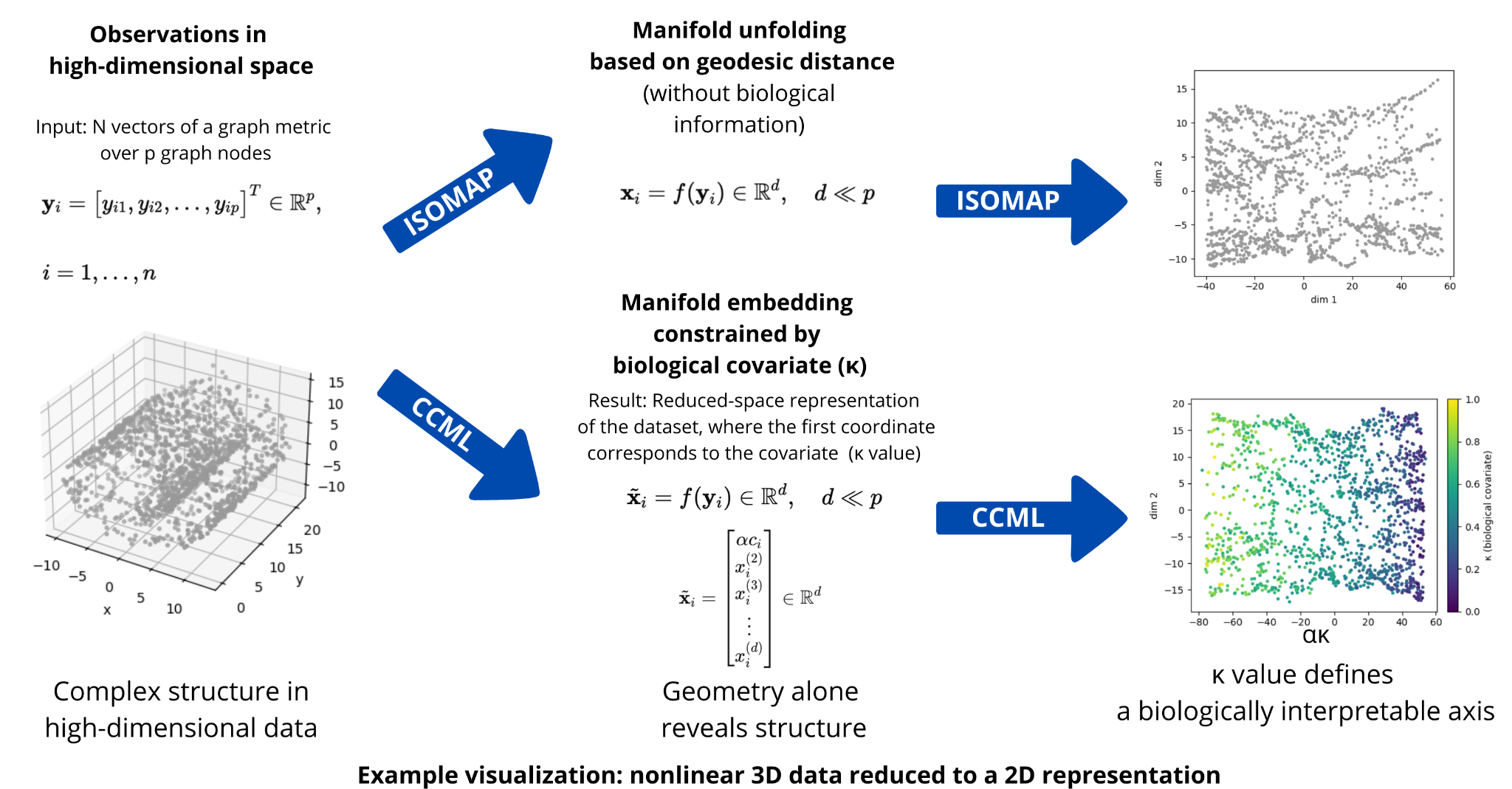


Results of the CCML analysis based on closeness centrality:


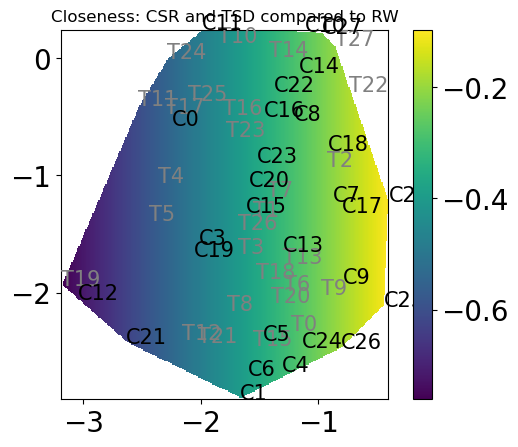


S11. Results for additional graph densities. Graphs were constructed at multiple edge densities by thresholding the correlation matrices to retain a fixed number of strongest connections. Specifically, we tested 15 edge thresholds ranging from 200 to 3000 edges (in steps of 200).

To confirm the robustness of our result, here we report results obtained using graphs constructed with 200 and with 600 edges. All statistical analyses were performed identically to those described in the main text of this publication.

Graph with 200 edges (5% cost):


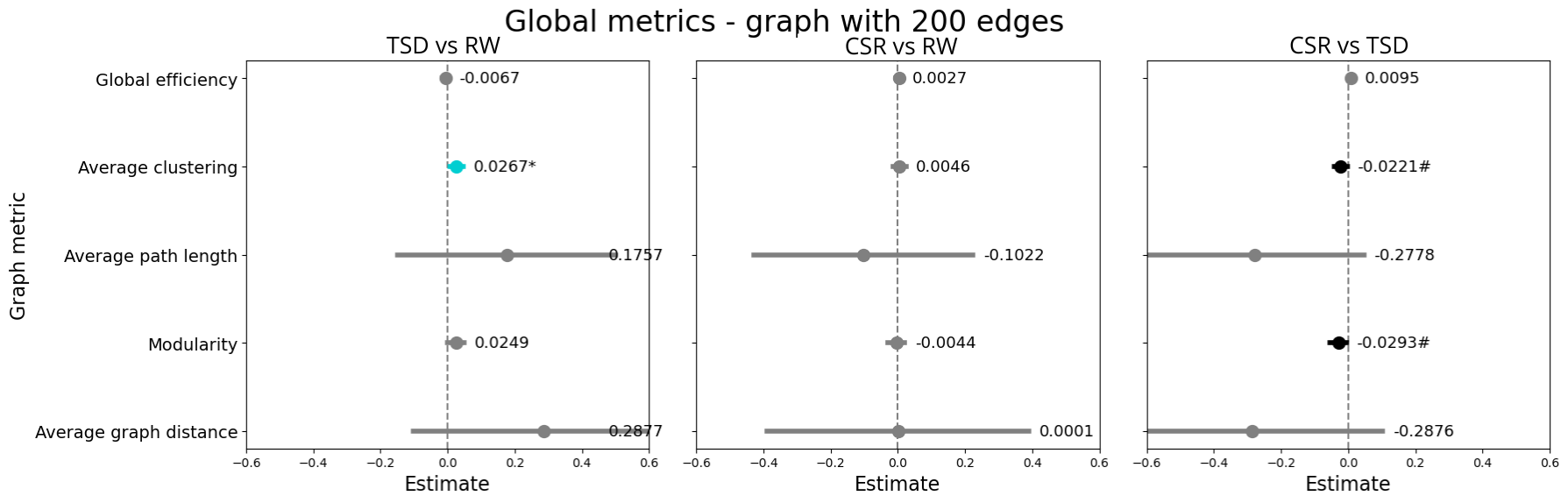


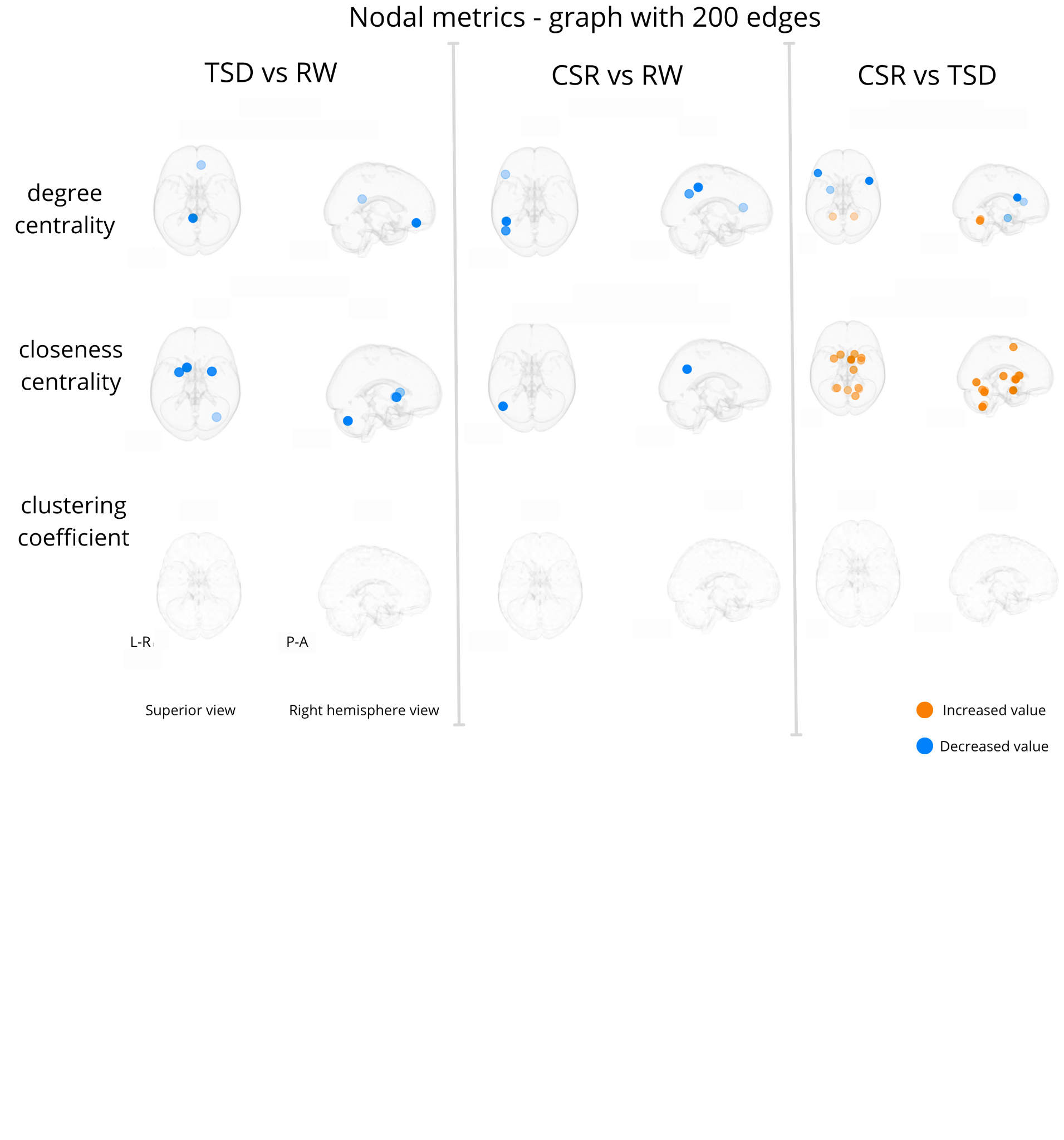


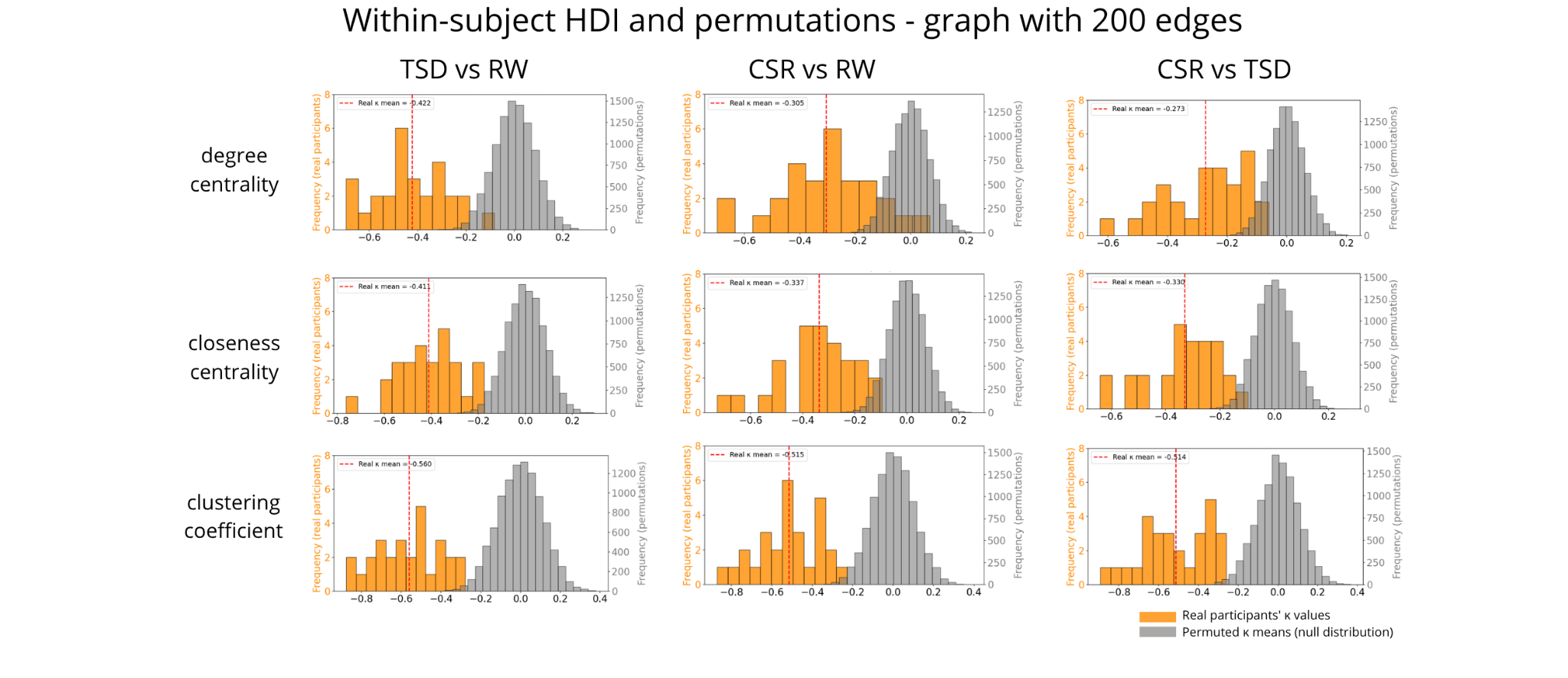


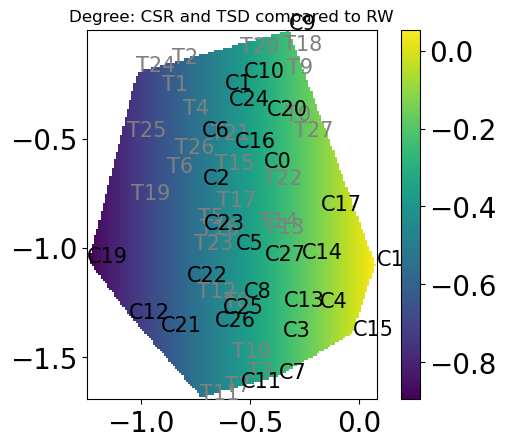


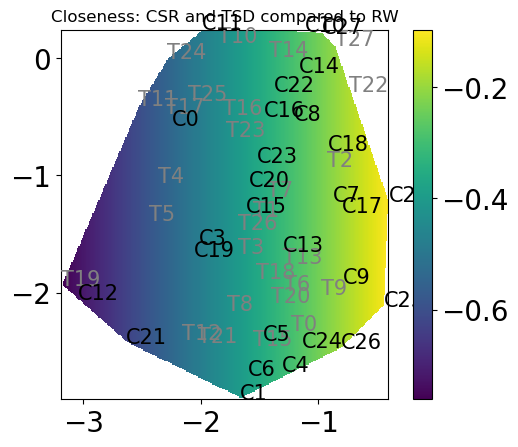


Graph with 600 edges (15% cost):


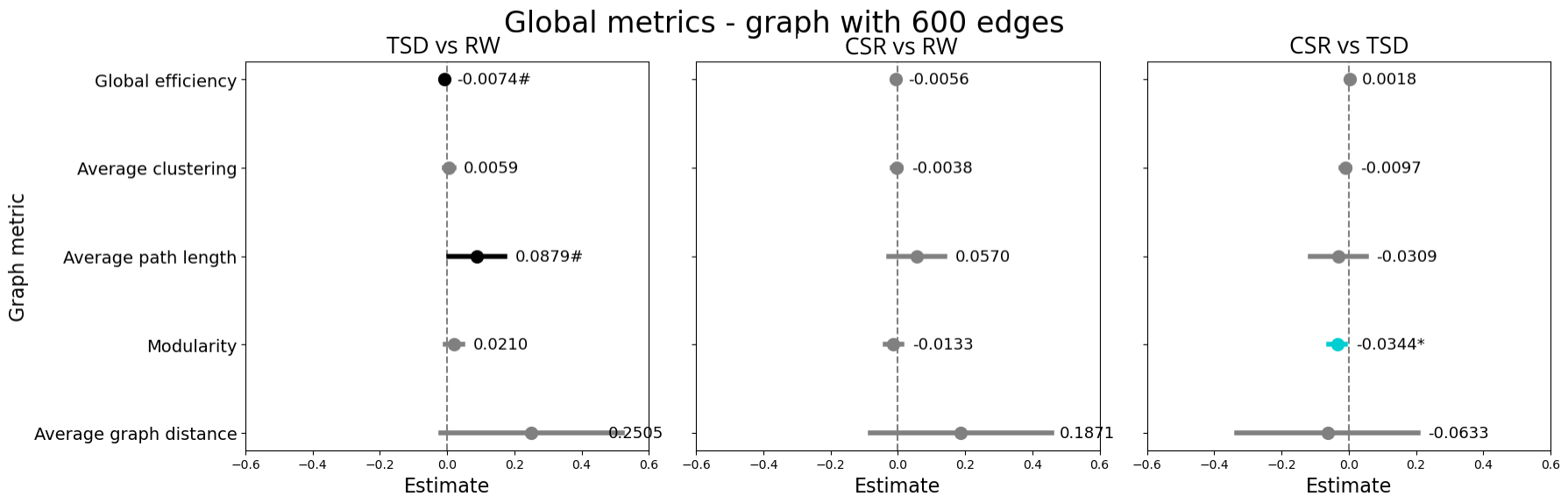


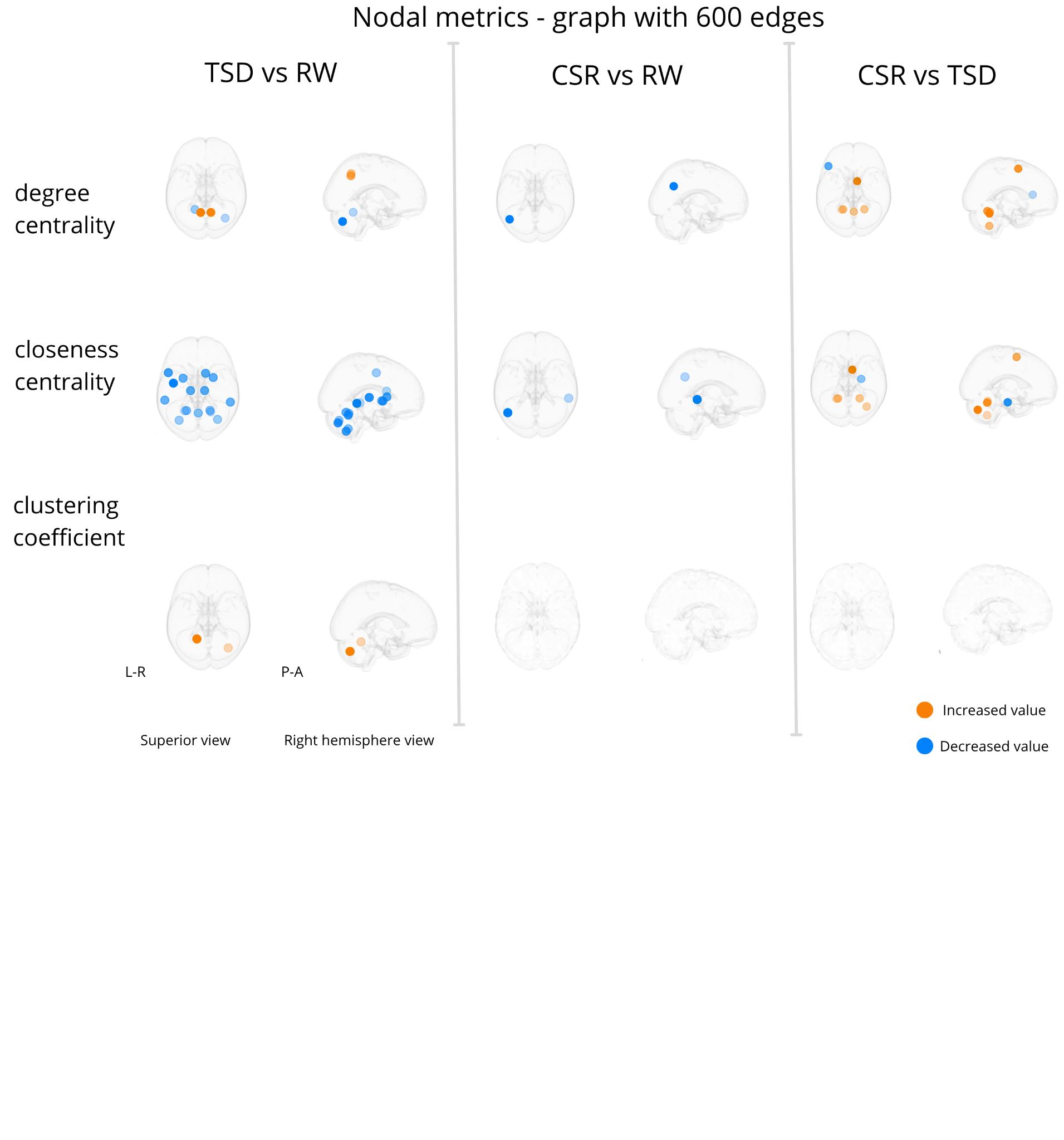


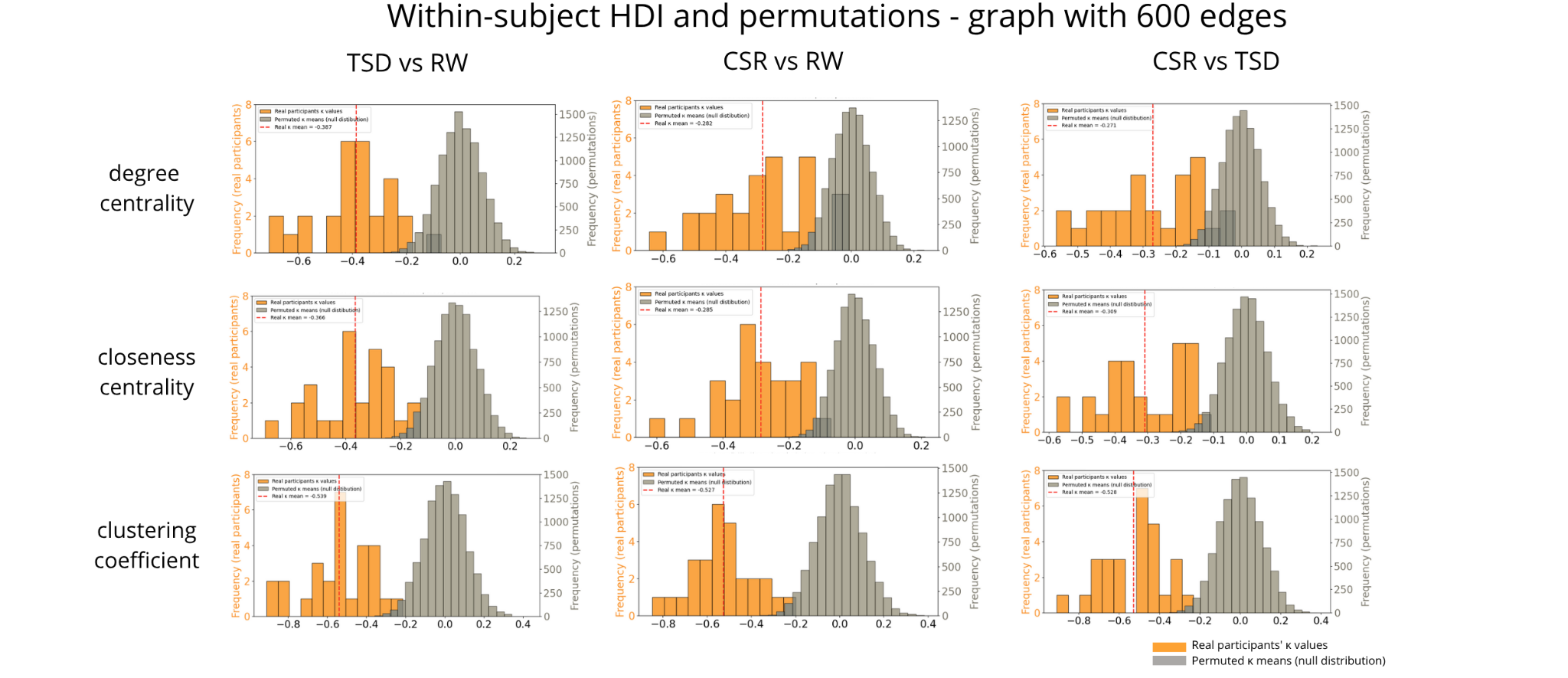


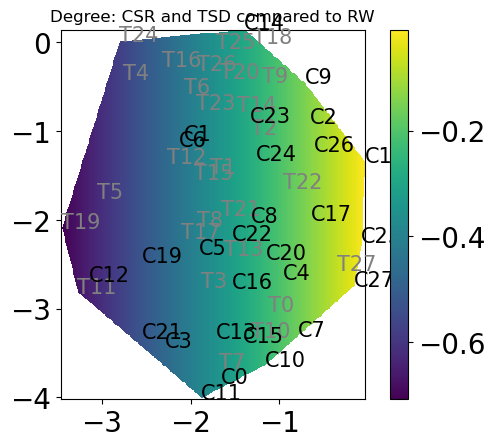


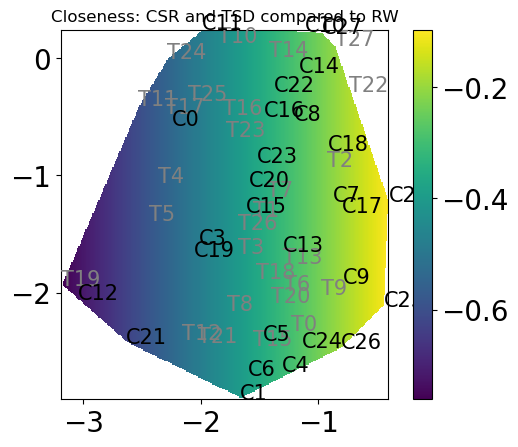


S12. Results for modified AICHA atlas (added cerebellum from AAL atlas) with 391 regions in total:


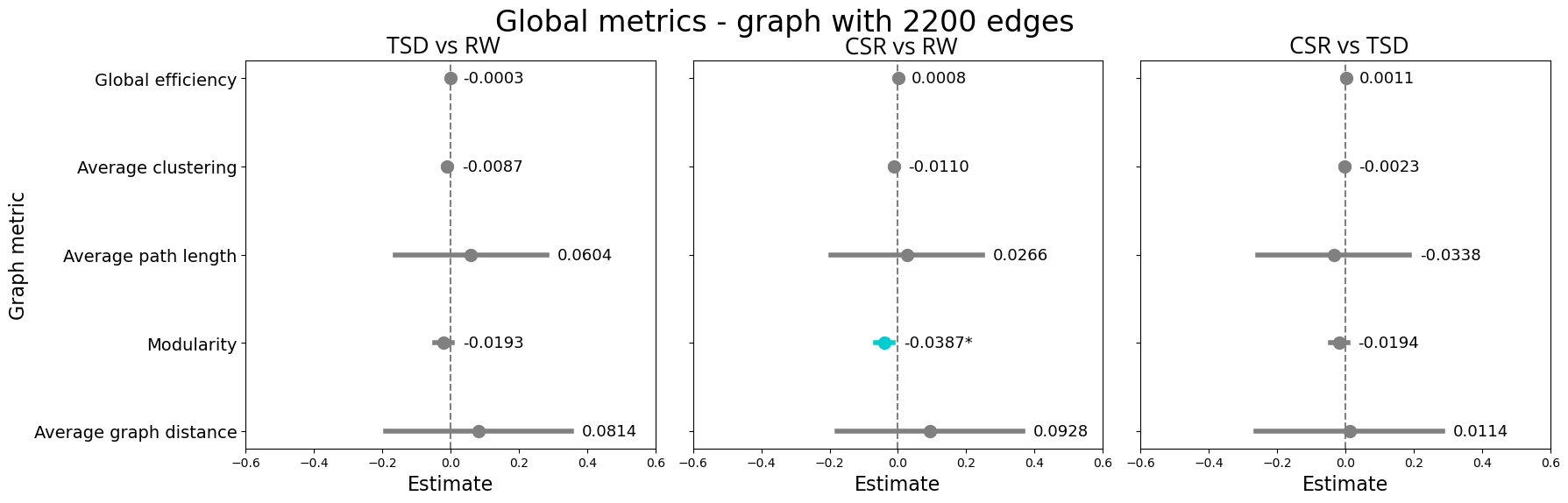


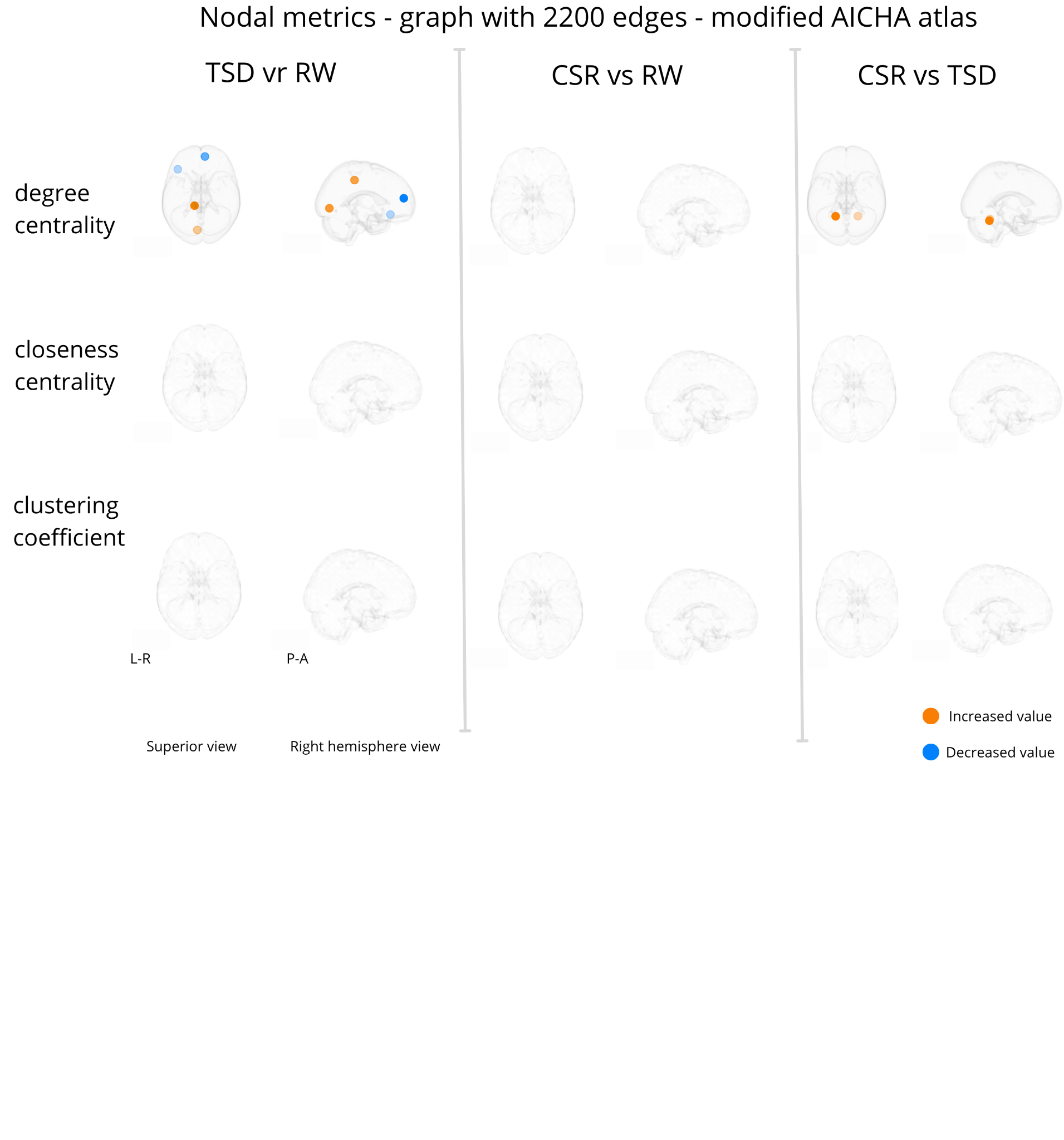


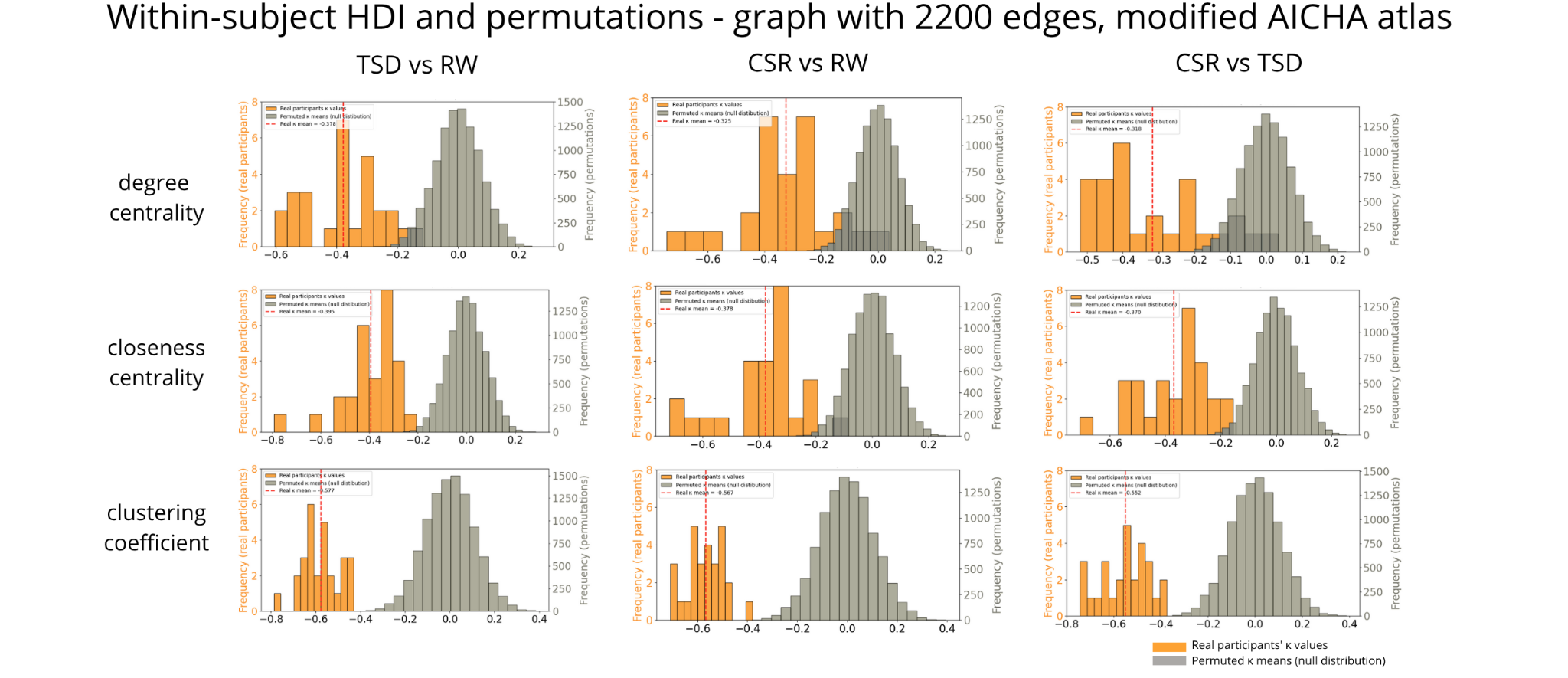


S13. Additional control within-subject HDI analyses using degree as a representative nodal metric under 3 alternative approaches:

1) weighted degree based on absolute correlation values with a MST,

2) weighted degree using the strongest absolute correlations without MST constraints,

3) binarized degree adjusted for differences in global mean correlation.


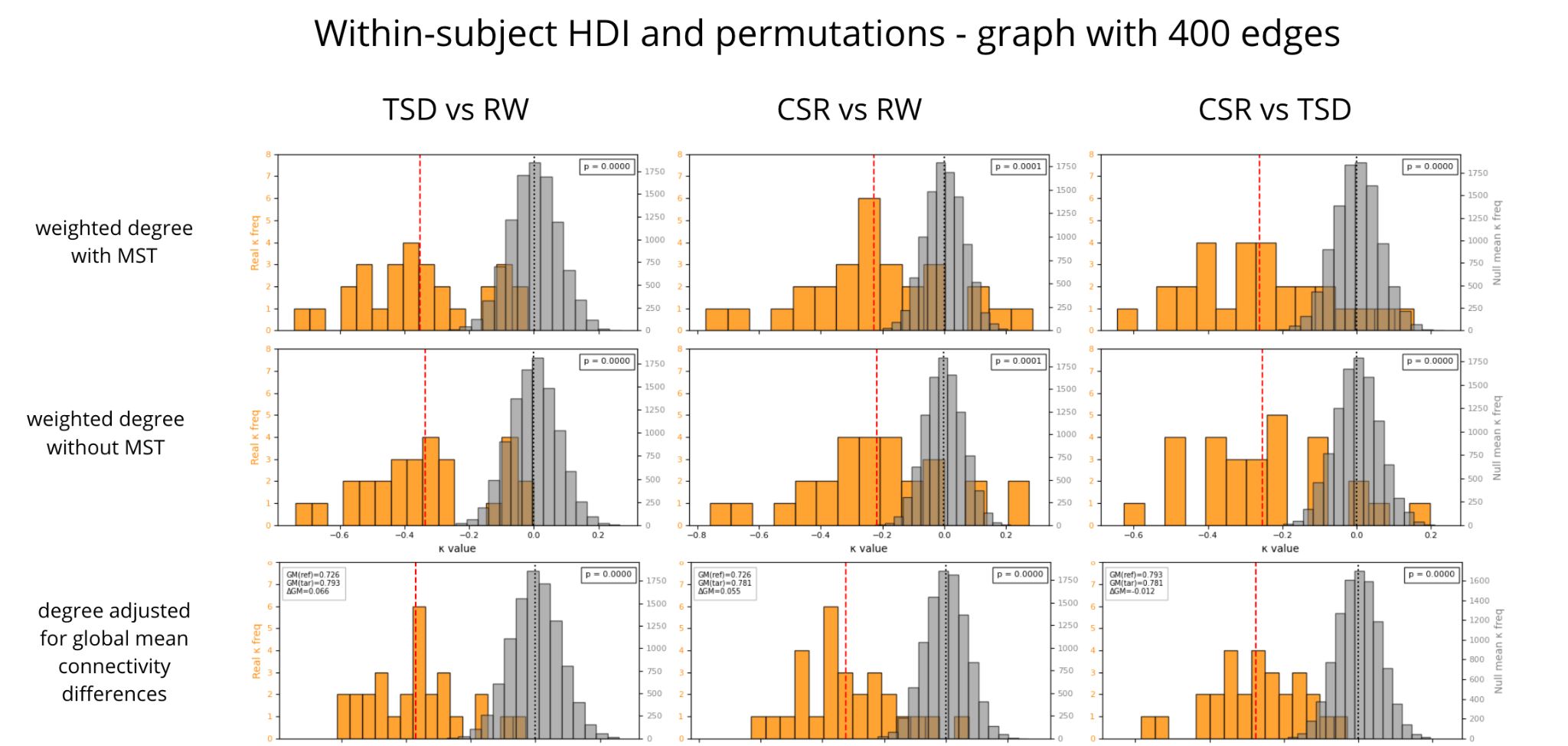


S14. Subjective sleep-related and circadian measures - descriptive statistics: mean, standard deviation, minimum value, quartiles Q1 (25%), median (50%), Q3 (75%), and maximum values.

|  | **Age** | **Morningness-Eveningness** | **Subjective amplitude of the circadian rhythm** | **Insomnia**  **Severity Index** | **Pittsburgh Sleep**  **Quality Index** | **Karolinska**  **Sleepiness**  **Scale - RW** | **Karolinska**  **Sleepiness**  **Scale - TSD** | **Karolinska  Sleepiness  Scale - CSR** |
| --- | --- | --- | --- | --- | --- | --- | --- | --- |
| mean | 23,57 | 22.79 | 18.25 | 5.75 | 4.61 | 3.04 | 6.26 | 5.29 |
| standard deviation | 3.72 | 4.35 | 4.74 | 1.94 | 1.59 | 1.69 | 2.10 | 1.67 |
| min. value | 20.00 | 15.00 | 11.00 | 1.00 | 1.00 | 1.00 | 1.00 | 1.00 |
| Q1 (25%) | 21.00 | 20.00 | 14.75 | 5.00 | 4.00 | 2.00 | 5.50 | 4.00 |
| median (50%) | 22.50 | 23.00 | 17.00 | 6.00 | 4.50 | 2.50 | 7.00 | 5.50 |
| Q3 (75%) | 25.00 | 26.25 | 23.00 | 7.00 | 6.00 | 3.25 | 8.00 | 6.25 |
| max. value | 36.00 | 30.00 | 26.00 | 11.00 | 7.00 | 7.00 | 9.00 | 8.00 |

S15. Stability of CCML/Isomap embeddings assessed via leave-one-out (LOO) and Support Vector Machine (SVM) analysis

Both LOO procedures demonstrated stable separability between TSD and CSR. When removing one participant from the original embedding, the centroid distances remained consistent with the full-sample value (~0.6-0.7 vs. 0.65). When recomputing a new embedding at each iteration, the distances formed a distribution, closely matching the original centroid distance (0.65), indicating that no single subject disproportionately influenced the manifold structure. The auxiliary SVM analysis achieved approximately 70% accuracy with balanced precision, recall, and F1 scores across conditions, further supporting that the embedding reflects meaningful sleep-related differences rather than overfitting or noise.

Leave-one-out distances computed on the original CCML embedding:


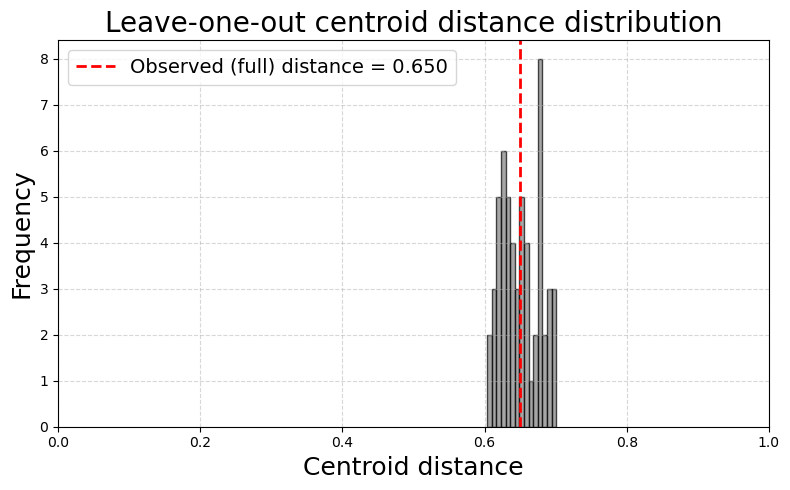


Leave-one-out centroid distances from refitted CCML embeddings (*n* = 27 per iteration):


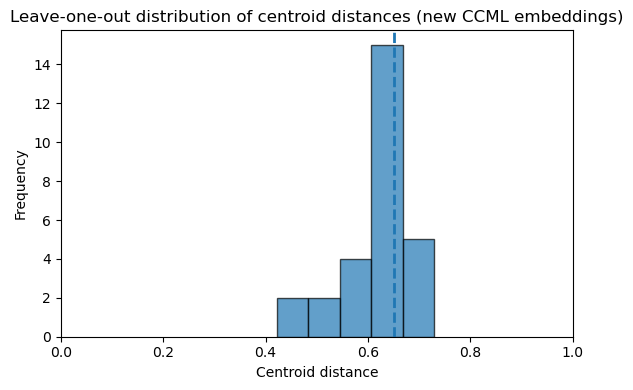


SVM classification performance for distinguishing TSD vs CSR based on CCML/Isomap embedding:

| **Metric** | **Value** |  |  |  |
| --- | --- | --- | --- | --- |
| **Accuracy per fold** | 0.75, 0.45, 0.91, 0.64, 0.73 |  |  |  |
| **Mean accuracy** | 0.70 |  |  |  |
| **Standard deviation** | 0.15 |  |  |  |
|  |  |  |  |  |
|  | **Predicted T** | **Predicted C** |  |  |
| **True TSD** | 20 | 8 |  |  |
| **True CSR** | 9 | 19 |  |  |
|  |  |  |  |  |
| **Class** | **Precision** | **Recall** | **F1-score** | **Support** |
| **TSD** | 0.69 | 0.71 | 0.70 | 28 |
| **CSR** | 0.70 | 0.68 | 0.69 | 28 |
| **Overall accuracy** | 0.70 |  |  |  |
| **Macro average** | 0.70 | 0.70 | 0.70 | 56 |
| **Weighted average** | 0.70 | 0.70 | 0.70 | 56 |

S16. Results of the linear Mixed-Effects Model. Our goal was to verify if the subjective sleepiness is associated with global graph metrics (global efficiency, average clustering coefficient, average shortest path length, modularity, and average graph distance) in each of our experimental conditions (RW, TSD, CSR).

| **Global metric** | **Condition** | ***β*** | **z** | **CI_low** | **CI_high** | ***p*-value FDR** |
| --- | --- | --- | --- | --- | --- | --- |
| Global efficiency | **RW** | **-0.0045** | **-2.6765** | **-0.0078** | **-0.0012** | **0.0223** |
|  | TSD | -0.0011 | -0.7797 | -0.0039 | 0.0017 | 0.5189 |
|  | CSR | 0.0011 | 0.6451 | -0.0022 | 0.0044 | 0.5189 |
| Average clustering | RW | 0.0001 | 0.0206 | -0.0066 | 0.0067 | 0.9836 |
|  | TSD | -0.0037 | -1.3247 | -0.0093 | 0.0018 | 0.4383 |
|  | CSR | -0.0031 | -1.0533 | -0.0088 | 0.0027 | 0.4383 |
| Average shortest path length | **RW** | **0.0673** | **02.6097** | **0.0168** | **0.1179** | **0.0272** |
|  | TSD | 0.0242 | 01.938 | -0.0192 | 0.0675 | 0.4111 |
|  | CSR | -0.0049 | -0.1874 | -0.0565 | 0.0467 | 0.8514 |
| Modularity | RW | 0.0031 | 0.4902 | -0.0092 | 0.0154 | 0.8494 |
|  | TSD | -0.0047 | -0.8930 | -0.0150 | 0.0056 | 0.8494 |
|  | CSR | -0.0013 | -0.1899 | -0.0142 | 0.0117 | 0.8494 |
| Average graph distance | **RW** | **0.1381** | **02.6410** | **0.0356** | **0.2405** | **0.0248** |
|  | TSD | 0.0250 | 0.5969 | -0.0572 | 0.1073 | 0.5880 |
|  | CSR | 0.0290 | 0.5417 | -0.0758 | 0.1337 | 0.5880 |

S17. Results of the OLS model. Our goal was to verify if trait-level sleep and circadian measures predict brain global graph metrics.

| **Global metric** | **Predictor** | ***β*** | **R^2^** | ***p*-value FDR** |
| --- | --- | --- | --- | --- |
| average clustering | AM | -0.0074 | 0.1665 | 0.5367 |
|  | ME | 0.0035 | 0.1665 | 0.6699 |
|  | PSQI | -0.0150 | 0.1665 | 0.2101 |
| average path length | AM | -0.0115 | 0.0135 | 0.8354 |
|  | ME | -0.0083 | 0.0135 | 0.8354 |
|  | PSQI | -0.0287 | 0.0135 | 0.8354 |
| average graph distance | AM | -0.1272 | 0.0664 | 0.6718 |
|  | ME | -0.0347 | 0.0664 | 0.6979 |
|  | PSQI | -0.0781 | 0.0664 | 0.6718 |
| global efficiency | AM | 0.0010 | 0.0258 | 0.7964 |
|  | ME | 0.0011 | 0.0258 | 0.7964 |
|  | PSQI | 0.0024 | 0.0258 | 0.7964 |
| modularity | AM | -0.0181 | 0.3456 | 0.0744 |
|  | ME | 0.0099 | 0.3456 | 0.2304 |
|  | **PSQI** | **-0.0356** | **0.3456** | **0.0009** |

S18. Results of the OLS model. Our goal was to verify if trait-level sleep and circadian measures predict differences in the functional connectivity between our experimental conditions.

| **Nodal metric** | **HDI comparison** | **Predictor** | ***β*** | **R^2^** | ***p*-value _FDR** |
| --- | --- | --- | --- | --- | --- |
| Degree centrality | κ_TSD_vs_RW | AM | 0.0189 | 0.0858 | 0.7897 |
|  |  | ME | -0.0434 | 0.0858 | 0.7897 |
|  |  | PSQI | -0.0112 | 0.0858 | 0.7897 |
|  | κ_CSR_vs_RW | AM | 0.0064 | 0.1519 | 0.8280 |
|  |  | ME | -0.0345 | 0.1519 | 0.5546 |
|  |  | PSQI | -0.0677 | 0.1519 | 0.1136 |
|  | κ_CSR_vs_TSD | AM | 0.0207 | 0.1894 | 0.6677 |
|  |  | **ME** | **-0.0635** | **0.1894** | **0.0211** |
|  |  | PSQI | -0.0149 | 0.1894 | 0.7089 |
| Closeness centrality | κ_TSD_vs_RW | AM | 0.0016 | 0.0729 | 0.9589 |
|  |  | ME | -0.0063 | 0.0729 | 0.9589 |
|  |  | PSQI | -0.0369 | 0.0729 | 0.8683 |
|  | κ_CSR_vs_RW | AM | 0.0280 | 0.1878 | 0.4848 |
|  |  | ME | -0.0141 | 0.1878 | 0.6155 |
|  |  | PSQI | -0.0519 | 0.1878 | 0.2463 |
|  | κ_CSR_vs_TSD | AM | 0.0404 | 0.2345 | 0.2251 |
|  |  | **ME** | **-0.0594** | **0.2345** | **0.0466** |
|  |  | PSQI | -0.0089 | 0.2345 | 0.8083 |
| Clustering Coefficient | κ_TSD_vs_RW | **AM** | **0.0946** | **0.3490** | **0.0094** |
|  |  | ME | 0.0076 | 0.3490 | 0.8902 |
|  |  | PSQI | 0.0049 | 0.3490 | 0.8902 |
|  | κ_CSR_vs_RW | **AM** | **0.0652** | **0.2096** | **0.0570** |
|  |  | ME | 0.0153 | 0.2096 | 0.9224 |
|  |  | PSQI | -0.0020 | 0.2096 | 0.9459 |
|  | κ_CSR_vs_TSD | AM | 0.0047 | 0.0084 | 0.9486 |
|  |  | ME | 0.0146 | 0.0084 | 0.9486 |
|  |  | PSQI | 0.0031 | 0.0084 | 0.9486 |
